# Capillary-Inserted Rotor Design for HRµMAS NMR-Based Metabolomics on Mass-Limited Neurospheres

**DOI:** 10.3390/molecules22081289

**Published:** 2017-08-03

**Authors:** Nghia Tuan Duong, Masanori Yamato, Masayuki Nakano, Satoshi Kume, Yasuhisa Tamura, Yosky Kataoka, Alan Wong, Yusuke Nishiyama

**Affiliations:** 1 Advanced Solid-State NMR Unit, RIKEN CLST-JEOL Collaboration Center, RIKEN, Yokohama, Kanagawa 230-0045, Japan; nghiatuan.duong@riken.jp; 2 Multi-Modal Microstructure Analysis Unit, RIKEN CLST-JEOL Collaboration Center, RIKEN, Kobe, Hyogo 650-0047, Japan; yamatomasa@riken.jp (M.Y.); satoshi.kume@riken.jp (S.K.); tamuray@riken.jp (Y.T.); kataokay@riken.jp (Y.K.); 3 Cellular Function Imaging Team, RIKEN Center for Life Science Technologies, Kobe, Hyogo 650-0047, Japan; cannakano@gmail.com; 4 Department of Physiology, Osaka City University Graduate School of Medicine, Abeno-ku, Osaka 545-8585, Japan; 5 NIMBE, CEA, CNRS, Université Paris-Saclay, CEA Saclay, 91191 Gif-sur-Yvette, France; alan.wong@cea.fr; 6 Engineering Division, JEOL RESONANCE Inc., Musashino, Akishima, Tokyo 196-8558, Japan

**Keywords:** NMR, metabolomics, metabolites, HRµMAS, capillary-inserted rotor, mass-limited neurospheres

## Abstract

Nuclear magnetic resonance (NMR) spectroscopy is a powerful analytical technique and has been widely used in metabolomics. However, the intrinsic low sensitivity of NMR prevents its applications to systems with limited sample availabilities. In this study, a new experimental approach is presented to analyze mass-scarce samples in limited volumes of less than 300 nL with simple handling. The sample is loaded into the glass capillary, and this capillary is then inserted into a Kel-F rotor. The experimental performance of the capillary-inserted rotor (capillary-insert) is investigated on an isotropic solution of sucrose by the use of a high-resolution micro-sized magic angle spinning (HRµMAS) probe. The acquired NMR signal’s sensitivity to a given sample amount is comparable or even higher in comparison to that recorded by the standard solution NMR probe. More importantly, this capillary-insert coupled with the HRµMAS probe allows in-depth studies of heterogeneous samples as the MAS removes the line broadening caused by the heterogeneity. The NMR analyses of mass-limited cultured neurospheres have been demonstrated, resulting in high quality spectra where numerous metabolites are unambiguously identified.

## 1. Introduction

Metabolomics is the qualitative and quantitative study of low weight molecules (metabolites) in biological systems, which enables a deeper understanding of the metabolic pathways of interest under defined conditions. The comprehensive analyses of such metabolites are achieved by the application of various analytical techniques, of which nuclear magnetic resonance (NMR) is a leading method, owing to its simultaneous acquisition of mixtures, simple sample preparation, quantitative and reproducible nature, and non-destructive characteristics [1,2,3,4,5,6,7,8]. Unfortunately, NMR is an intrinsically insensitive technique, thus a large amount of sample is required for acquiring interpretable spectra. This sensitivity issue restricts the application of NMR from systems with limited sample availabilities.

Tremendous efforts have been made to improve the NMR sensitivity of mass/volume-limited samples. The most obvious approach is to employ a higher magnetic field since the sensitivity improves with the field strength, but this is not an economical selection because the doubling of the field requires significantly higher cost. Another solution for this issue is the use of a micro coil (µcoil) together with tiny sample holders. However, a sample volume of less than one microliter (µL) is challenging due to the magnetic susceptibility effect, which severely reduces the resolution, making µcoil insufficient for metabolomic investigations. The spectral broadening caused by the discontinuity of magnetic susceptibility within the sample can be removed by magic angle spinning (MAS), namely high-resolution MAS (HRMAS) or field-gradient MAS (FGMAS). However, these probes typically required 20–50 µL samples with rotors of 3.2 mm to 4.0 mm in diameter and are not suitable for mass-scarce samples. Moreover, these large rotors suffer from temperature gradients due to frictional heating even at moderate (2–5 kHz) MAS rates, resulting in additional broadening in temperature-dependent peaks. A great advance for NMR-based metabolomics for mass/volume-limited samples is the introduction of the inductively coupled high-resolution magic angle coil spinning method (HRMACS) [9,10,11,12,13,14]. In the innovative work, the tiny secondary coil is in close proximity to, and is spun with the sample holder. The advantages are: (i) higher radio frequency efficiency and optimal filling factor (the fraction of the sample with the detection coil volume), thus achieving higher sensitivity, and (ii) the removal of the magnetic susceptibility broadening from the µcoil and the sample heterogeneity, yielding high resolution up to 0.002 ppm [12]. This technique managed to characterize the metabolic profiles of intact cells and whole tiny organisms. Despite its wide applications in metabolomic analysis, the mechanical work for operating the µcoil of HRMACS is challenging, leading to the concern of reproducibility after the replicate acquisitions. Furthermore, the sample spinning is limited to avoid frictional and Eddy current [15] heating, resulting in the incomplete elimination of the anisotropic interactions. Hence, the spinning sidebands suppression experiment must be applied to acquire isotropic spectra for the analysis.

A further advancement for simplifying the labor work of HRMACS is the modified µMAS probe (HRµMAS), where no µcoil manipulation is required [16,17]. In this experimental design, the use of (i) copper–aluminum as coil material for better matching with the magnetic susceptibility of air, and (ii) the Kel-F (polychlorotrifluoroethylene) rotor instead of zirconia, as well as the replacement of zirconia air-bearings inside the MAS stator by Vespel offered sufficient resolution for the study of metabolomic profiling of animal organs, such as liver and brain [17]. Nevertheless, a number of limitations should be considered. Firstly, as the Kel-F rotor is tiny and light, the spinning frequency is unstable and limited, making the obtained spectra hard to interpret. Secondly, a great deal of care is required for sample packing to avoid any samples, dusts, and/or solvents on the surface of the rotor to facilitate the sample spinning. Therefore, the packing procedure is time-consuming, approximately half an hour. Such a lengthy process can affect the final results since metabolites degrade with time. Thirdly, the occurrence of air bubbles in the rotor not only distorts the internal magnetic field lines (thus affecting the spectral resolution), but also prevents an accurate quantitative analysis of the sample of interest. Finally, the cost-ineffectiveness is also a concern. As made by the soft material, Kel-F rotors are fragile to manipulation during the sample preparation process and cannot be used even with minor distortions.

For overcoming the difficulties caused by the Kel-F rotor and as a continuation of our previous works [17], herein we present a new rotor design by using a glass capillary as a sample holder, which is then placed into the Kel-F rotor. In this article, we will test the practical performance of the capillary-inserted Kel-F rotor (capillary-insert) coupled with the HRµMAS probe on the isotropic solution of sucrose, and then investigate the feasibility of this setup for studying the NMR-based metabolomics for heterogeneous neurosphere samples with tiny quantities.

## 2. Experimental

### 2.1. Sample Preparation

For sucrose, the 0.5 M solution was prepared by dissolving 172.0 mg of sucrose in 1.0 mL D_2_O. The solution of 2.5 mM sucrose used for our experiments was prepared by diluting the 0.5 M solution.

For producing neurospheres, all experimental protocols were approved by the Ethics Committee on Animal Care and Use of the RIKEN Center for Life Science Technologies (MAH22-04-5), and were performed in accordance with the Principles of Laboratory Animal Care. Brain tissue was taken from rats following application of a physical insult [18]. Neurospheres were obtained from the tissue by the conventional floating culture method [19]. Neurospheres with diameters of 100–240 μm were taken from culture dishes using a micropipette, and then culture medium was washed with 10.0 mM phosphate-buffered saline (PBS). After centrifugation (1000 g for 5 min at room temperature), the supernatant was discarded and then washed with physiological saline to remove phosphorus. Then, some samples were treated with 500 μL of methanol for removing the proteins, which simplifies NMR spectra since these broad peaks hinder the unambiguous identification of metabolites. All samples with/without methanol were concentrated by evaporation of methanol and/or physiological saline during a cooled centrifugation for 1 h. Two types of neurosphere samples (methanol-treated and non-treated) underwent the NMR analyses. Despite the existence of two types of neurospheres, the experimental procedure for both is identical. The samples were prepared from the unfiltered supernatant of swelled neurospheres, which were mixed with 6–8 µL D_2_O and then stirred gently. This volume of D_2_O was optimized to well dissolve the entire sample and to offer as high concentration of metabolites as possible. The solution was immediately used after preparation to minimize the degradation of metabolites.

### 2.2. Rotor Packing Procedure

**Figure 1:**
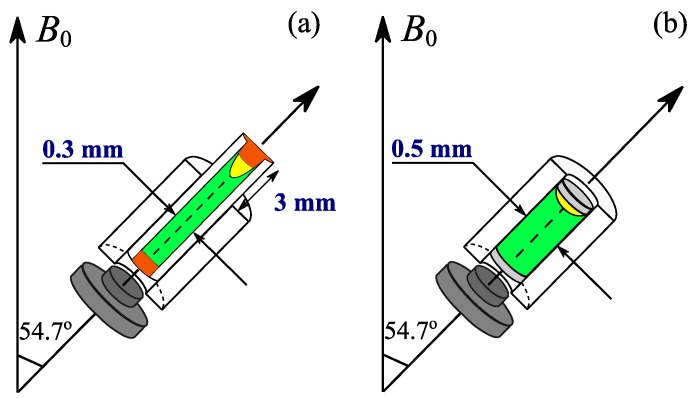
Illustration of the new capillary-inserted rotor (**a**) and the previous Kel-F rotor (**b**) designs. In (**a**), the sample (green) is contained in the glass capillary where the two ends are sealed by wax (orange). The sealed capillary is located inside the Kel-F rotor. In (**b**), the sample (green) is directly injected into the rotor and then enclosed by the two Kel-F inserts (light grey). The small gaps (yellow) between the wax (**a**) or the Kel-F insert (**b**) and the sample of the two models refer to air bubbles, which are outside or inside of the detection region, respectively. The extension length (3 mm) of capillary in (**a**) is used for better visualization, so it is not correctly scaled.

In order to be employed for this newly designed rotor, the capillary should tightly fit to the existing Kel-F rotors with high concentricity. In addition, the wall thickness of the capillary should be thick enough so that it is not fragile, but is thin enough to maximize the sample volume. These requirements are met by the design of the capillaries with the outer diameters of 0.500 ± 0.020 mm and the inner diameters of 0.300 ± 0.015 mm, with the length of 300 mm (Fuji-Rika-Kogyo, Osaka, Japan). Despite its high precision, the capillary needs to be pre-screened for good matching with the specific Kel-F rotor. To facilitate the sample insertion and removal, the 7 mm capillary was prepared so that it extends approximately 3 mm above the Kel-F rotor. With such an approach, air bubbles (if any) would stay at the tip of the capillary, thus being out of the detection volume of approximately 280 nL. Only a bottom cap is required for this configuration, since the other end of Kel-F rotor should be open for the extended glass capillary. As the Kel-F rotor is designed longer than the conventional 1 mm zirconia rotor, the driving gas is directly applied to the rotor surface rather than turbine cap which is absent in the current system. This enables slow sample spinning of several kilohertz with high stability since the spinning is less sensitive to driving pressure fluctuation. Upon loading the sample of interest, the capillary was sealed by quickly immersing its tips into melted wax. This aims to prevent sample leakage during spinning. Then, the sealed capillary, followed by the bottom cap were gently inserted into the rotor. The visualization of the capillary-inserted rotor is shown in Figure 1a.

Unlike the previous Kel-F rotor in Figure 1b, the entire procedure of this new design was mostly performed on the capillary, thus it keeps the rotor surface clean, helping to avoid spinning failure. More importantly, this procedure removes the demanding task of sealing the rotor with two tiny inserts. These noticeable differences provide a number of experimental advantages, which will be discussed in the following section.

### 2.3. NMR Spectroscopy

All the NMR experiments were performed at the room temperature of 25 °C, using a JNM-ECZ600R spectrometer (JEOL RESONANCE Inc., Akishima-shi, Tokyo, Japan) with 14.1 T of the magnetic field, corresponding to a ^1^H Larmor frequency of 599.67 MHz. Solution NMR experiments were collected using the ROYAL probe with the room temperature coil. The solvent signal suppression was performed with delay alternating with nutation for tailored excitation (DANTE) [20] presaturation pulses during the repetition delay. HRµMAS experiments were carried out on a modified µMAS probe, and under no ^2^H lock condition. The MAS frequency was set between 1.2–1.5 kHz, and the recycle delay was set to 5.0 s. The *B*_0_ shimming was optimized on a solution of sucrose-D_2_O sample, and this optimal shimming profile was also used for other experiments. The lengths of π/2 and π pulses on the ^1^H channel were 1.0 and 2.0 µs, respectively. For the sucrose sample, the single pulse experiment was employed while for the neurosphere samples, in order to minimize the effect of lipid and fatty acid peaks, were recorded with Carr–Purcell–Meiboom–Gill (CPMG) sequence [21]. The residual H_2_O solvent signal suppression sequence was performed by using a weak continuous wave (CW) presaturation pulse during the recycle delay. For recording the spectra, 32 K data points were collected within a spectra width of 20 ppm, corresponding to the maximum achievable resolution of 0.6 ppb. The line-broadening of 0.5 Hz was applied to all obtained spectra for data analysis. The ^1^H chemical shifts were based on the internal lactate methyl doublet at δ^iso^ = 1.33 ppm. Further experimental details will be given in the figure captions.

## 3. Results and Discussion

This section is categorized into two parts. The first part examines the practicality and performance of the capillary-insert on sucrose sample, while the second part investigates the feasibility of this new rotor design on studying mass-limited neurosphere samples.

### 3.1. Practicality and Performance of the Capillary-Inserted Rotor Design

The glass capillary-insert exhibits practical advantages over the Kel-F rotor in these aspects: (i) *Rotor contamination*. Since the involvement of the Kel-F rotor in the entire sample packing procedure is minimized, this approach avoids the risk of any samples, dusts, and/or solvents on the rotor surface, hence facilitating the sample spinning; (ii) *Sample preparation*. Owing to the elimination of the most time-consuming steps of Kel-F inserts, sealing and rotor cleaning, the packing process is shortened to less than 10 min. Not only is the laboratory work simplified, but also degradation of metabolites is minimized; (iii) *Sample destruction*. As the sample is only contained in the capillary, the centrifugal force exerted on it is trivial because this force is proportional to the inner diameter of the capillary, which is 0.3 mm. This is an essential point for delicate biological samples; (iv) *Sample exchange and quantification*. To study new samples, the capillary needs to be replaced while the same Kel-F rotor is being used. With such extended length (Figure 1a), the capillary can be simply exchanged. Moreover, it is crucial that the quantitative results are feasible because the air bubbles do not affect the detection region, as mentioned in Section 2.2; (v) *Cost-effectiveness*. The glass capillaries are inexpensive and ready for insertion and removal from the Kel-F rotor. This also allows the sample holder (capillary) to be disposable, which is important in clinical applications.

Despite the aforementioned advances, sensitivity is an inevitable limitation of the capillary-insert method. The filling factor is smaller in the case of the capillary-insert since the sample stays further away from the detection coil (Figure 1a). In addition, the detection volume within the capillary (Figure 1a) is less than that of the Kel-F rotor (Figure 1b), namely 280 nL versus 490 nL, respectively. Therefore, lower NMR signals of the capillary-insert are expected. It should be kept in mind that there is a trade-off between the ease of rotor packing and the sensitivity.

**Figure 2:**
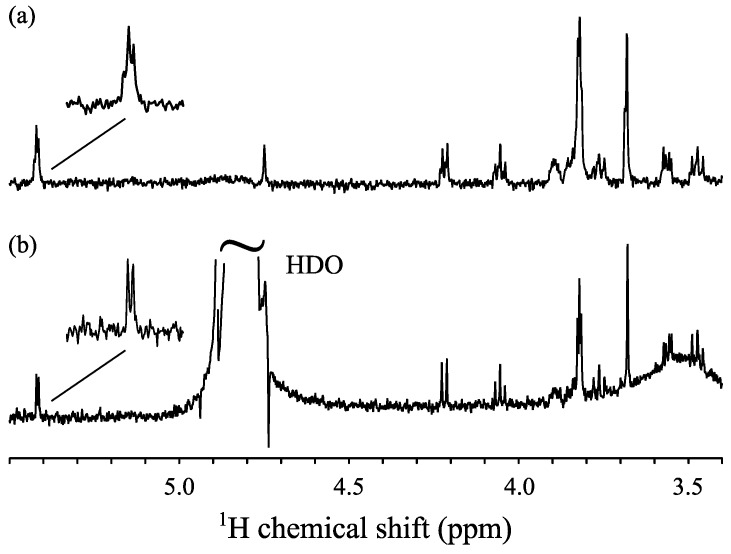
The 1D proton spectra of 0.25 µg sucrose recorded by ^1^H single pulse HRµMAS probe using a capillary-insert (**a**) and by solution NMR using a conventional 5 mm tube (**b**). 1024 scans were collected. Each experiment took for 1.5 h.

As the capillary-insert shows practical superiorities compared to the previous Kel-F rotor, the next question is whether this new setup could provide sufficient resolution for studying metabolomics. This is investigated by comparing the 1D spectra of a sucrose solution acquired by ^1^H single pulse HRµMAS and solution NMR. Indeed, sucrose solution is an isotropic solution, hence no MAS is required and standard solution NMR can be used. For the HRµMAS probe experiment, the capillary was filled with the 2.5 mM sucrose solution. As the detection volume is 280 nL, it corresponds to 0.25 µg of sucrose within the detecting region. For the solution NMR experiment, the 2.5 mM sucrose solution was diluted so that same amount of 0.25 µg sucrose was contained in a 600 µL detection volume of the conventional 5 mm NMR tube. The results are demonstrated in Figure 2.

It is apparent that the solution NMR spectrum in Figure 2b provides higher resolution, but all the main features of peaks as well as their splittings of the sucrose sample are demonstrated in Figure 2a. For example, for the peak at ~5.4 ppm or the peak at ~3.6 ppm, the doublet or doublet of doublets are clearly observed, respectively. Besides providing a similar spectrum, HRµMAS also offers the following three advantages. First is the comparable or even higher intensity, which is essential for the study of mass/volume-limited samples. Different from the evident peak at 3.9 ppm in Figure 2a, the same peak in Figure 2b is hardly observed, owing to the equivalent intensity of the noise level. It is noted that the peaks from Figure 2a are broader than those in Figure 2b. The broadening is owing to the limitations of the hardware as well as the imperfect shimming. This drawback may affect the data analysis for isotropic metabolite solutions, however, it is less problematic for systems where the intrinsic resolution is limited due to sample heterogeneity. Second is the cleaner spectrum. While the residual water peak is well suppressed in Figure 2a, this peak is prominent at 4.7–4.9 ppm in Figure 2b, which could hinder the observation of other peaks surrounding. For solution NMR, the water suppression is not sufficient since the amount of solvent used is considerably larger than that of the sample of interest. This is in contrast to the HRµMAS experiments where a greatly reduced amount of expensive deuterated solvent was used, thus better water suppression was achieved. It should be noted the sophisticated water suppression sequences for solution NMR could further remove the residual signal, however, we prefer a simple sequence to assure reproducibility. As less amounts of solvents were used, the spectrum is less interfered with by the solvent impurities, as well as by possible contaminations on the surface of the sample tube that give a broad signal at 3.5 ppm in Figure 2b. Although these signals can be removed in solution NMR by using highly pure solvents together with careful sample tube treatment, similar or even higher quality spectra can be easily obtained by HRµMAS without great attention. The better water suppression capability is more crucial in tissue samples where no D_2_O can be applied and huge residual H_2_O signal is expected. Third is the ease of experimental processing. Unlike solution NMR, where the shimming profile is optimized from sample to sample, only minor or even no further shimming adjustment is required for each sample because of MAS averaging of susceptibility broadenings. This is beneficial for metabolomics NMR in terms of reproducibility and robustness towards sample degradation. In short, this capillary-inserted rotor design simplifies the sample preparation with low-cost operation.

The results from Figure 2 illustrate the sufficiency of the capillary-insert with the HRµMAS probe for acquiring high-resolution spectra from a tiny amount of a sucrose sample. The next step is to use this experimental setup with the identical shimming profile from sucrose for metabolite analyses of samples of mass-limited neurospheres. It is worth noting that the same Kel-F rotor was used for the capillary-insert, which assures the optimal shimming conditions for all subsequent experiments.

### 3.2. Feasibility of the Capillary-Inserted Rotor on Studying Mass-Limited Neurosphere Samples

**Figure 3:**
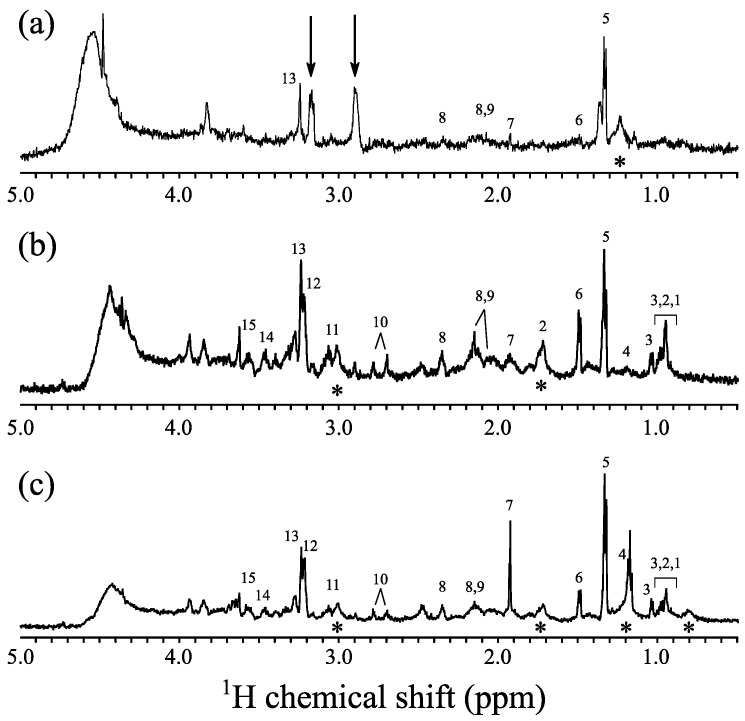
Proton 1D spectra of methanol treated (**a**) and untreated (**b**,**c**) neurosphere samples. All experiments were recorded by Carr–Purcell–Meiboom–Gill (CPMG) sequence with the total echo delay of 1.0 ms. The experimental times were from 15 to 20 h. The asterisk (*) denotes the lipid peak. The detailed assignments are given in Table 1.

For metabolomic analysis, the three ^1^H-NMR spectra of the neurosphere samples (one treated and two untreated with methanol), recorded by the use of capillary-insert HRµMAS, are shown in Figure 3.

The samples were prepared from the unfiltered supernatant of swelled neurospheres, hence, they inevitably show a certain degree of heterogeneity due to small contamination of tissues. Although this introduces additional broadening in the standard solution NMR experiments, this broadening is largely removed under the MAS condition. Hence, HRµMAS resulted in three well-resolved spectra where various metabolites are identified and their J-splitting could be measured. For example, the doublet of peaks of valine, lactate, and alanine at 1.02, 1.33 and 1.46 ppm, respectively, are clearly observed. As shown here, the resolution limitation of the HRµMAS probe is less problematic in real samples due to residual broadening originating from sample heterogeneity.

While for the treated sample in Figure 3a, there are only six metabolites are unambiguously assigned, the number of metabolites for untreated neurospheres are fifteen, as shown in Figure 3b,c. This result is in contrast to our expectation, since with the methanol treating, the proteins that obscure the observation of metabolites are removed, and a simplified spectrum is obtained, therefore, more or at least a similar number of peaks are expected. One possible explanation of fewer peaks might be due to the methanol treatment resulting in the removal of metabolites. The difference in the number of metabolites evidently distinguishes the treated and untreated neurosphere samples. It is noted that in Figure 3a, there are two visible peaks at 2.9 ppm and 3.2 ppm, highlighted by the two arrows; we have no explanation for the occurrence of these peaks at present. Despite the differences in peak heights (especially significant changes for 3-hydroxybutyrate (peak number 4) and acetate (peak number 7) at 1.2 and 1.9 ppm, respectively), the spectra of the two untreated neurospheres in Figure 3b,c reveal the same number of metabolites (15).

The significant advantage of using the capillary-insert with the HRµMAS probe is that it could readily carry out single neurosphere NMR investigations. As each sample, consisting of twenty neurospheres, was diluted in 6–8 µL of D_2_O, in which the detection region for capillary is only 280 nL, the neurosphere count within this active volume is less than one unit. This suggests the possibility of individual specific analysis of neurosphere samples.

In general, with this new rotor design combined with a HRµMAS probe, high-quality spectra of single neurospheres can be acquired for mass-limited samples. Moreover, based on the identified metabolites, it is possible to distinguish the treated and untreated neurosphere samples.

## 4. Conclusions

This work highlights the considerable progress in rotor design, in which the glass capillary is placed into the rotor. This capillary-inserted rotor shows practical superiorities compared to Kel-F rotors in terms of sample preparation, spinning stability, and cost-effectiveness, despite the sacrifice of sensitivity. Regarding the experimental performance, the use of a capillary-insert coupled with the HRµMAS probe offers more simplified spectra as less solvent is used, leading to better suppression of any residual water signal. More importantly, this enables individual neurosphere analysis for mass-limited samples. Based on the number of unambiguously assigned metabolites, two types of neurospheres are evidently distinguished. The capillary-insert and HRµMAS probe could open a new way for the comprehensive analysis of metabolites, and offer significant advantages in a wide range of applications where high throughput of mass-limited samples is essential [23].

## Figures and Tables

**Table 1:** Proton chemical shift assignments of metabolites from 1D spectra of neurosphere samples from Figure 3 [22].

Number	^1^H Shift (δ) (ppm)	Molecule
1	0.93 (t); 1.00 (d); 1.28 (m); 1.47 (m); 1.96 (m)	isoleucine
2	0.95 (d); 0.97 (d); 1.71 (m); 3.69 (dd)	leucine
3	0.97 (d); 1.02 (d); 2.24 (m); 3.57 (d)	valine
4	1.20 (d); 2.31 (m); 2.38 (m); 4.13 (m)	3-hydroxybutyrate
5	1.33 (d)	lactate
6	1.46 (d); 3.76 (q)	alanine
7	1.91 (s)	acetate
8	2.00 (m); 2.14 (m); 2.36 (m)	glutamate
9	2.08 (m); 2.09 (m); 2.41 (m); 3.68 (t)	glutamine
10	2.68 (dd); 2.81 (dd)	aspartate
11	2.89 (t); 2.96 (t); 3.01 (t)	lysyl
12	3.19 (s); 3.50 (m)	phosphoryl choline
13	3.24 (s); 3.67 (dd); 3.78 (m)	glycerol phosphocholine
14	3.25 (t); 3.41 (t)	taurine
15	3.28 (t); 3.56 (dd); 4.06 (t)	myo-inositol

Abbreviation: s = singlet; d = doublet; t = triplet; dd = doublet of doublets; q = quartet; and m = multiplet.
